# Whole Exome Sequencing and Panel-Based Analysis in 176 Spanish Children with Neurodevelopmental Disorders: Focus on Autism Spectrum Disorder and/or Intellectual Disability/Global Developmental Delay

**DOI:** 10.3390/genes15101310

**Published:** 2024-10-11

**Authors:** Ariadna Sánchez Suárez, Beatriz Martínez Menéndez, Eduardo Escolar Escamilla, Francisco J. Martínez Sarries, Miren Iranzu Esparza Garrido, Belén Gil-Fournier, Soraya Ramiro León, Bárbara Rubio Gribble, Juan F. Quesada Espinosa, Andrés J. Alcaraz Romero

**Affiliations:** 1 Escuela Internacional de Doctorado, Rey Juan Carlos University, Alcorcón Campus, 28922 Madrid, Spain; 2 Faculty of Biomedical and Health Sciences, Universidad Europea de Madrid, Villaviciosa de Odón, 28670 Madrid, Spain; bmmenendez@salud.madrid.org (B.M.M.); eduardo.escolar@salud.madrid.org (E.E.E.); barbara.rubio@salud.madrid.org (B.R.G.); aaromero@um.es (A.J.A.R.); 3 Neurology Department, Getafe University Hospital, 28905 Madrid, Spain; fmsarries@salud.madrid.org; 4 Pediatrics Department, Getafe University Hospital, 28905 Madrid, Spain; iranzuesparza@hotmail.com; 5 Genetics Department, Getafe University Hospital, 28905 Madrid, Spain; belen.gilfournier@salud.madrid.org (B.G.-F.); soraya.ramiroleon@salud.madrid.org (S.R.L.); 6 Genetics Department, 12 de Octubre University Hospital, 28041 Madrid, Spain; juanf.quesada@salud.madrid.org

**Keywords:** neurodevelopmental disorders, whole exome sequencing, autism spectrum disorder, intellectual disability, diagnostic yield, variants of uncertain significance

## Abstract

Background: Neurodevelopmental disorders (NDDs) represent a significant challenge in pediatric genetics, often requiring advanced diagnostic tools for the accurate identification of genetic variants. Objectives: To determine the diagnostic yield of whole exome sequencing (WES) with targeted gene panels in children with neurodevelopmental disorders (NDDs). Methods: This observational, prospective study included a total of 176 Spanish-speaking pediatric patients with neurodevelopmental disorders (NDDs), encompassing intellectual disability (ID), global developmental delay (GDD), and/or autism spectrum disorder (ASD). Participants were recruited from January 2019 to January 2023 at a University Hospital in Madrid, Spain. Clinical and sociodemographic variables were recorded, along with genetic study results. The age range of the subjects was 9 months to 16 years, and the percentage of males was 72.1%. The diagnostic yield of whole exome sequencing (WES) was calculated both before and after parental testing via Sanger DNA sequencing. Results: The study included 176 children: 67 (38.1%) with ID, 62 (35.2%) with ASD, and 47 (26.7%) with ASD + ID. The diagnostic yield of proband-only exome sequencing was 12.5% (22/176). By group, the diagnostic yield of proband-only exome sequencing was 3.2% in the ASD, 12.7% in the ASD + ID, and 20.8% in the ID group. Variants of uncertain significance (VUS) were found in 39.8% (70/176). After parental testing, some variants were reclassified as “likely pathogenic”, increasing the diagnostic yield by 4.6%, with an overall diagnostic yield of 17.1%. Diagnostic yield was higher in patients with syndromic ID (70.6%% vs. 29.4%; *p =* 0.036). Conclusions: A sequential approach utilizing WES followed by panel-based analysis, starting with the index case and, when appropriate, including the parents, proves to be a cost-effective strategy. WES is particularly suitable for complex conditions, as it allows for the identification of potentially causative genes beyond those covered by targeted panels, providing a more comprehensive analysis. Including parental testing enhances the diagnostic yield and improves accuracy, especially in cases with variants of uncertain significance (VUS), thereby advancing our understanding of NDDs.

## 1. Introduction

Neurodevelopmental disorders (NDDs) constitute a diverse set of conditions, including intellectual disability (ID), global developmental delay (GDD), and autism spectrum disorder (ASD).

According to the Diagnostic and Statistical Manual of Mental Disorders [1], ASD is a developmental disorder characterized by persistent deficits in social communication and interaction, alongside restricted and repetitive patterns of behavior.

Global developmental delay (GDD) refers to a condition characterized by significant delays in achieving age-appropriate developmental milestones in two or more domains, such as motor skills, speech and language, cognition, social interaction, and daily living activities. This diagnosis is typically reserved for children under the age of five, as a precursor to a more specific diagnosis like intellectual disability (ID), once the child is older and more accurately assessed [2].

According to the DSM-5, intellectual disability (ID) is diagnosed based on significant deficits in both intellectual and adaptive functioning. These deficits must occur during the developmental period (before age 18) and affect the individual’s ability to perform everyday activities. Intellectual functioning is typically assessed using standardized tests such as the Wechsler intelligence scale for children (WISC) or similar tools, with an IQ score below 70 indicating the presence of ID [1].

NDDs, affecting an estimated 2–3% [3,4,5] of newborns, are at the forefront of contemporary medical and social debates. These diseases are highly heterogeneous, involving a complex interplay of genetic and environmental factors that contribute to their varied manifestations [6,7].

Etiological diagnosis by identifying genes associated with disease can improve the understanding of pathogenesis and prognosis of NDDs. Nevertheless, it is essential to ascertain the percentage of individuals with NDDs who carry a genetic abnormality by using next-generation sequencing (NGS) [8]. The diagnostic yield refers to the proportion of exome analysis cases with variants classified as pathogenic or likely pathogenic that correlate with the patient’s phenotype. Recent studies have found that exome sequencing (ES) could diagnose anywhere from 8% to 61% [9,10,11] of undiagnosed cases of NDDs: 3–28% of cases of ASD and 28–43% of cases of GDD and/or ID [12,13,14,15].

The rapid advancement of ES techniques, together with trio-based analysis, now leads to diagnostic yields of up to 50%. Trio-based ES was recently proposed as the most effective molecular diagnostic approach, but its high cost limits its application [16,17]. An alternative is a sequential approach beginning with ES of the proband (the affected individual) and followed by parental verification via Sanger/NGS [18].

WES has become significantly more affordable compared to five years ago, making it a highly competitive option in genetic testing today. The future of genetics will likely involve genome analysis and long-read sequencing technologies, which promise to enhance our understanding of complex genetic conditions. While gene panels are effective for well-characterized genes and provide faster analysis, they can also be designed to target intronic and non-coding regions, which may play critical roles in disease causation. Recent studies underscore the significance of variants in non-coding regions, highlighting their potential impact on gene regulation and expression. For instance, one study demonstrates how intronic variants can affect splicing and lead to functional consequences in gene activity [19]. Another article discusses the emerging role of non-coding variants in the pathogenesis of various disorders, providing evidence that these regions are not merely “junk” DNA but are integral to understanding genetic disease mechanisms [20]. Recognizing the importance of these variants is essential for a comprehensive approach to genetic testing and research.

In the current study, we prospectively evaluated 176 pediatric patients with NDDs, including ASD and/or ID/GDD. Our objective was to first calculate the diagnostic yield using WES through targeted gene panels in the proband, followed by calculating the diagnostic yield after incorporating a parental study.

## 2. Materials and Methods

### 2.1. Participants and Clinical Information

We carried out a prospective, observational, and analytical study that included pediatric patients between the ages of 0 to 16 years, who attended the Pediatric Neurology Department, University Hospital of Getafe, over a four-year period between January 2019 and January 2023.

The inclusion criteria were as follows: (1) diagnosis of NDDs, which included ID/GDD and/or ASD; (2) parental consent for genetic testing and result disclosure; (3) ES testing through targeted gene panels; (4) no findings in prior genetic (CGH Array, Fragile X) or metabolic tests; and (5) absence of other non-genetic causes of NDDs.

The diagnosis of NDDs followed the DSM-5 definitions [1]. Patients with GDD presented impairments in at least two developmental domains: fine and gross motor skills, language, social skills, cognitive function, and daily living activities. Based on clinical indications, certain patients had undergone prior diagnostic evaluations: array CGH; Fragile X testing or karyotyping; metabolic assessments; brain MRI, video-Electroencephalogram; or neuropsychological evaluation. In some cases, following ES in the proband, the genetics and neuropediatric team considered it necessary to conduct parental segregation studies of the variant found in their child to assess its causality.

**Figure 1:**
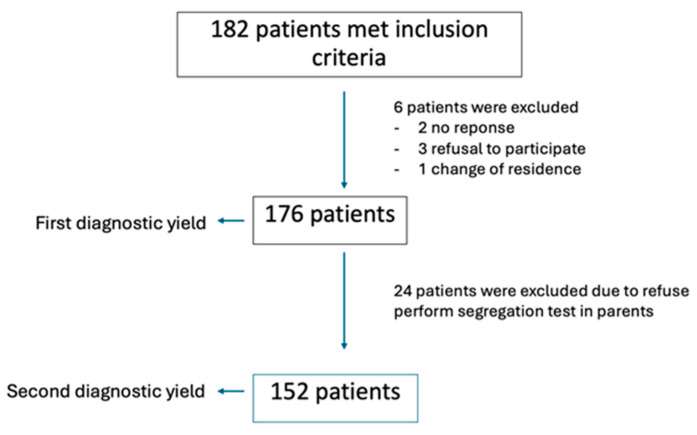
Flowchart.

A total of 182 patients met the inclusion criteria, but 6 patients were excluded for different reasons (see flowchart; Figure 1). The remaining 176 pediatric patients (127 males and 49 females) underwent exome sequencing, and the diagnostic yield was calculated. A total of 24 families did not accept parental testing, and for this reason, to calculate the diagnostic yield after parental testing, we only included the 152 families that accepted parental segregation testing.

### 2.2. Ethical Statement and Consent

This study was approved by the Ethics Committee of the University Hospital of Getafe. Informed written consent was obtained from the parents or legal guardian of each patient prior to enrollment. The authors have stated that they had no interests which might be perceived as posing a conflict or bias.

### 2.3. Data Collection

The collected variables included the following: (1) sociodemographic: age, sex, and ethnicity; (2) family history: similar phenotype in parents, consanguinity, family history of neurodevelopmental disorder, and age of parents at birth of their child; (3) patient’s clinical history: type of pregnancy (spontaneous or assisted reproduction), prenatal alterations, and neonatal pathology; (4) physical examination: weight, height and head circumference, and dysmorphic features; (5) clinical features: degree of ID (mild, moderate, or severe), psychomotor development, congenital anomalies, associated neurodevelopmental disorders (language disorders, learning difficulties, ADHD, and epilepsy); (6) cognitive: general intelligence assessed using the Wechsler intelligence scale for children (WISC-V), which evaluates a range of cognitive skills, including verbal comprehension, visual–spatial skills, fluid reasoning, working memory, and processing speed [21], and other aspects assessed include vocabulary, social cognition (emotion recognition, empathy), executive functions (working memory, inhibition, and cognitive flexibility), and attention; (7) diagnosis: result of exome sequencing, type of variant, inheritance pattern, genetic studies on parents, age at diagnosis, karyotype, array CGH, and metabolic studies.

### 2.4. Whole Exome Sequencing

The exome capture and enrichment was performed with xGen Exome Panel v1.0 (IDT—Integrated DNA Technologies, San Diego, NJ, USA). Paired-end sequencing (2 × 75 bp) of the whole exome was performed with the NextSeq 550 sequencing system from Illumina.

Bioinformatics analysis was conducted with the MatSeq tool, a homemade pipeline that integrates the programs BWA (v0.7.17-r1188) and Bowtie2 (v2.3.4) for sequence alignment against the reference genome (hg19 assembly), GATK (Haplotype Caller from Genome Analysis Toolkit, v.4.1) and VarDict (AstraZeneca, v1.7.0) for genotyping, ExomeDepth R package (v1.10) for CNV identification, and Annovar (v2018Apr16) and VEP (Ensembl, v105) for variant annotation. MatSeq meets the recommendations of validation established by the Association for Molecular Pathology and the College of American Pathologists.

### 2.5. Sanger DNA Sequencing

With this technique, the sequence flanking the variant under study is obtained. Based on the segregation result, an attempt will be made to reclassify the variants as probably causal or probably non-causal.

Automated DNA collection was performed on a Maxwell^®^ 16 System using the Maxwell^®^ 16 LEV Blood DNA kit (Promega, Madison, WI, USA). Quantification of the DNA was obtained by a spectrophotometry in a Biophotometer by measuring the absorbance at a wavelength of 260 nm. PCR amplification was conducted for the corresponding exon (+/−20 bp) and adjacent intronic regions of the gene to be studied. Sequencing of both strands of the amplified fragments and visualization of the sequences using capillary electrophoresis were carried out in an Applied Biosystems 3500DX Genetic Analyzer (Thermo Fisher Scientific, Waltham, MA, USA). Comparison of the sequences was obtained with a reference consensus sequence (GenBank Accession Number: NM_00088.4) for the corresponding gene, and the obtained results were interpreted.

### 2.6. Variant Inclusion and Classification

The analysis and interpretation of the variants identified in the genes of interest were performed according to the information in several resources and databases: gnomAD, ClinVar, HGMD, Uniprot, OMIM, Orphanet, GeneReviews, etc.

The analysis and interpretation of the variants identified in the genes of interest were performed according to the information in several resources and databases, with some obtained through automated annotation with VEP (Ensembl Variant Effect Predictor, v105) (gnomAD, v2.1; ClinVar, 20180930; Uniprot, Release 2019_01) and others through web site consultation (OMIM, https://www.omim.org, accessed on 1 October 2024; Orphanet, https://www.orpha.net, accessed on 1 October 2024; GeneReviews, https://www.ncbi.nlm.nih.gov/books/NBK1116, accessed on 1 October 2024. In silico predictions of variant impacts were obtained through automated annotation with VEP and dbNSFP (v4.0a) and were used in the interpretation and classification of variants according the ACMG criteria (PP3, BP4) [22].

The classification of the variants was performed according to the recommendations established by the ACMG based on the information extracted from the resources consulted and the assistance of semi-automatic classification tools like Varsome, Franklin, or Intervar.

### 2.7. Statistical Analysis

A descriptive analysis of data was performed using commercially available software (SPSS-PC, version 23.0; IBM Inc., Chicago, IL, USA). Data were expressed as frequencies and proportions or as medians and interquartile ranges (p25–p75). Categorical variables were compared using chi-square tests (with Fisher’s exact test when necessary), and continuous variables were compared using Mann–Whitney U test. Statistical significance was defined as *p* value of less than or equal to 0.05.

## 3. Results

A total of 182 patients met the inclusion criteria. The remaining 176 pediatric patients (127 males and 49 females) underwent exome sequencing, and the diagnostic yield was calculated.

**Figure 2:**
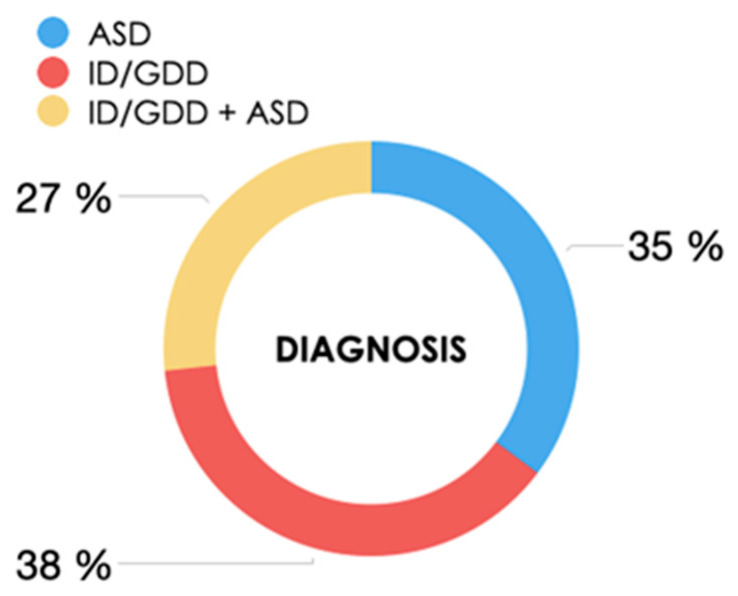
Classification of patients by diagnostic subgroups. ID: intellectual disability; ASD: autism spectrum disorder; and GDD: global developmental delay.

By diagnostic groups, 35.2% were diagnosed with ASD (62/176), 38.1% with GDD or ID (67/176), and 26.7% (47/176) with both (Figure 2).

Patients’ age ranged from 9 months to 16 years (median age of 6 years; interquartile range of 3–9 years). The male/female ratio revealed a higher proportion of males (72.1%), and the majority were of Caucasian origin (79%) (Table 1).

Consanguinity was present in 3.4% of cases, and 18.8% of patients had a first-degree relative with a history of intellectual disability. A total of 6.8% patients were conceived through assisted reproductive techniques. With regard to the type of delivery, we classified the patients in two groups: Group one was delivered by natural spontaneous delivery or scheduled cesarean section (77.2%). Group two was delivered by instrumental delivery or emergency cesarean section (22.8%). Average gestational age was 38 +/− 2 weeks. A total of 12% patients were preterm deliveries (4 newborns born at less than 32 weeks and 17 between 32 and 37 weeks).

**Figure 3:**
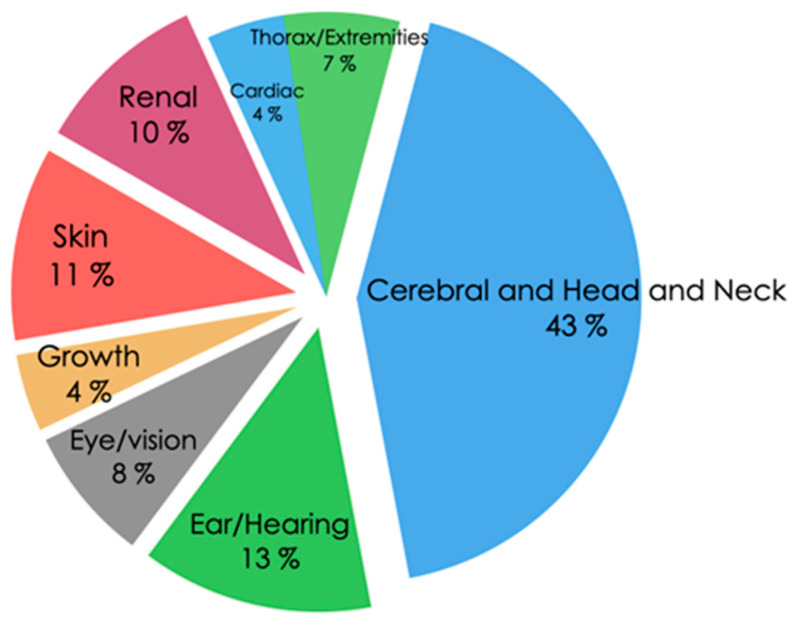
Dysmorphic features/congenital abnormalities. The frequency of dysmorphic features/congenital anomalies detected in the patients is described.

Dysmorphic features and congenital abnormalities were observed in 36.4% of the cases: 43% cerebral/head and neck, 13% ear/hearing, 11% skin, 10% renal, 8% eye/vision, 7% thorax/extremities, 4% growth, and 4% cardiac (Figure 3).

**Figure 4:**
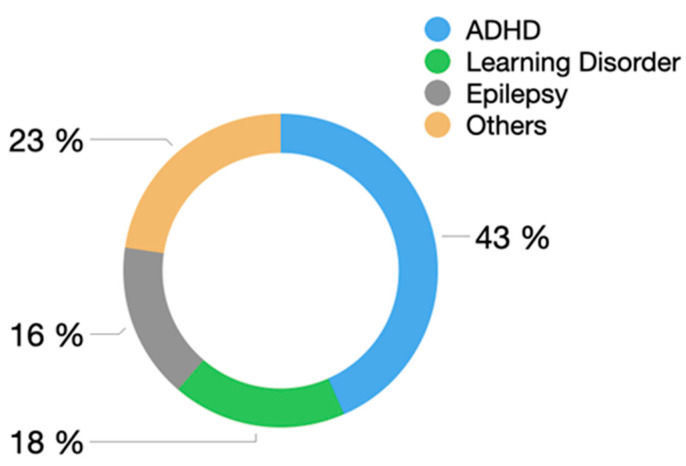
Associated comorbidity in patients. ADHD: attention deficit hyperactivity disorder.

We also analyzed comorbidity with other neurodevelopmental disorders: 26.1% patients also had ADHD, 10.8% learning disorders, 9.7% epilepsy, and 13.6% other conditions (sleep disorder, conduct disorders, tics/Tourette’s disorder, or coordination disorder) (Figure 4).

Additional genetic tests were conducted previously or simultaneously in some cases. The karyotype was performed in 44.8% of cases, revealing alterations in only one case. Array CGH was conducted in 67.7% of cases, with alterations found in nine patients.

X chromosome fragility study was performed in 78.9% of patients with alterations. A study of X chromosome fragility was performed in 78.9% of patients, with abnormalities identified in two cases. The first case involved a 9-year-old male diagnosed with intellectual disability and autism spectrum disorder (ASD), whose Fragile X test revealed a premutation (61 repeats). Whole exome sequencing (WES) uncovered a variant of uncertain significance (VUS) in the GRIN2A gene (c.4322C>T, p.T1441I), which was inherited from his asymptomatic mother.

The second case concerned a 15-month-old female presenting with traits of ASD and microcephaly. Her Fragile X test showed an intermediate allele (38 repeats). WES revealed a heterozygous VUS in the SETBP1 gene (c.776_777delinsTT, p.Ser259Phe), which was also inherited from her asymptomatic mother.

Pathogenic variants were identified in 5.1% of patients (9/176), probably pathogenic variants in 7.3% (13/176), and VUS in 48.9% (86/176). No pathological genetic findings were observed in 38.6% of patients (68/176).

The diagnostic yield was defined as the sum of patients with pathogenic and likely pathogenic variants relative to the total number of variants. Diagnostic yield by proband exome sequencing was 12.5% (22 of 176) and by groups was 3.2% in ASD, 12.7% in ASD + ID/GDD, and 20.8% in ID/GDD. 

After proband exome sequencing, in some cases, the parental segregation test was proposed via Sanger DNA sequencing in order to better characterize the possible causality of the detected variants, mainly in VUS and in some likely pathogenic variants that did not clearly match the patient’s phenotype.

It was deemed necessary to perform parental segregation studies in 90 patients, of which only 66 families agreed. After completing the parental segregation, we decided to calculate a second diagnostic yield, considering the variants that had been reclassified as likely causal or non-causal. However, this time we considered a total of 152 patients, after excluding the 24 families that did not agree to the familial segregation.

As a result, our diagnostic rate after the segregation test increased from 12.5% (22/176) to 17.1% (26/152), representing an additional 4.6% in diagnostic yield. Diagnostic yield by diagnostic groups increased to 23% in ASD + ID/GDD (up from 12.7%) and to 26.6% in ID/GDD (up from 20.8%). However, in the ASD group, the diagnostic yield decreased to 1.8% (down from 3.2%), as seen in Table 2.

In the ASD + ID/GDD group, our study showed a diagnostic yield of 66.7% in patients older than 5 years compared to 33.3% in younger children. However, these differences were not statistically significant (66.7% vs. 33.3%, *p =* 0.060), possibly due to the small sample size.

A wide genetic heterogeneity was observed, with variants identified in the following genes: HUWE1, KDM6B, SOX5, ASH1L, CIC, CUL3, HIVEP2, ZBTB18, CHAMP1, CNKSR2, MED13L, POGZ, NFIX, CDKL5, SPAST, DDX3X, DOCK8, GATA3, TLK2, SHANK3, CACNA1C, and CDH23 (Table 3). Chromosomal CNVs were identified in six cases (three deletions and three duplications) involving chromosomes 2, 7, 9, 16, and 22. The size of affected regions ranged from 529 Kb to 1.9 Mb (Table 4).

Notably, 11.9% of patients (21/176) had incidental findings unrelated to the initial reason for testing. These findings included actionable genes associated with hereditary cancer in 2 patients (*BRCA2* and *BRIP1* genes) and carrier states of autosomal recessive (AR) diseases in 19 patients (*GBA*, *PAH*, *TG*, *PKDH1*, *ACADM*, *USH2A*, *PMM2*, *ATP6V0A2*, *PRKN*, *ALDOB*, *DHCR7*, *IDUA*, *AHI1*, *XPC*, *PTS*, *SLC17A5*, and *RNASEH2B* genes).

The diagnostic yield of ES was analyzed based on different variables according to their diagnosis: ASD, ASD + ID/GDD, or isolated ID/GDD.

For the ASD group, no statistically significant differences in diagnostic rate were found, according to different variables. However, for the ASD + ID/GDD and ID/GDD groups, significant differences were observed when congenital anomalies and dysmorphic features were present (*p* = 0.010 and *p* = 0.036, respectively). No significant differences were found based on sex, age, maternal age (whether > or <35 years), ethnicity, consanguinity, first-degree family history of disease, or the degree of intellectual disability.

## 4. Discussion

NDDs are a significant health concern, affecting over 3% of children worldwide. Achieving an NDD diagnosis is challenging due to substantial clinical and genetic heterogeneity [6].

Whole exome sequencing (WES) is recommended as the first-tier test for NDDs, with trio sequencing being ideal for detecting de novo variants. However, cost constraints have led to the adoption of a sequential study [23,24].

We obtained an overall diagnostic yield of 17.1% (26/152), which is lower than what has been reported in published studies [10,11,12,13,14,15]. In the isolated ID/GDD group, our diagnostic yield was 26.6% (16/60), and in the ID/GDD + ASD group, it was 23% (9/39), both being closer to findings in other studies.

We present data from 176 Spanish pediatric patients who underwent ES using genetic panels in their diagnostic process. Identifying the genetic basis for a child’s neurodevelopmental condition can provide essential insights into their prognosis.

Furthermore, genetic studies enable healthcare providers to offer more precise recurrence risk counseling for families, aiding in informed reproductive decision making [25].

The present study explores the use of clinical WES in probands, followed by parental analysis in selected cases, as a cost-effectiveness alternative in the etiological diagnosis of NDDs.

A notable finding in our study is the lower diagnostic yield compared to other studies. A recent systematic meta-analysis of 30 NDD genetic testing studies reported a clinical diagnostic yield of 36% for individuals with NDDs. Specifically, the molecular diagnostic yield was 16% for primarily ASD cases, 39% for primarily ID cases, and 39% for the heterogenous group of ID and/or ASD [12].

One possible reason for our lower diagnostic yield could be that other studies include probands along with parents or families. Our study also included targeted panel sequencing studies, while previous meta-analyses on NDDs focused on ES. Stefanski et al. performed a systematic review comparing genetic testing yields across NDD subtypes and sequencing technologies. They stratified study cohorts by sequencing technology (ES, n = 36; panels, n = 73), finding a diagnostic yield of 27.2% for ES (95% CI = 24–31%) and 22.6% for panels (95% CI = 20–25%) [5], although the difference was not statistically significant (27.2% vs. 22.6%, *p =* 0.071). However, in previous systematic meta-analyses, this difference was more pronounced [12].

It is difficult to estimate how parental segregation analysis for all families would have affected the diagnostic yield. In our study, we had to exclude 24 families who declined to undergo the segregation study. If this had not occurred, the number of VUS would likely have been reduced, thereby increasing the final diagnostic yield [26,27].

In the isolated ASD group, our yield was 1.8% (1/53), which is lower than that reported in other studies. Over the study years, we observed an increase in the number of patients under 5 years old referred to child neurology department for possible language delay or GDD and/or ASD. Many of these children subsequently showed favorable progress in their neurodevelopment.

Arteche-López et al. discuss the appropriate age for offering genetic testing to patients with ASD, which remains a controversial issue. In their study, 84% (37/44) of patients with a genetic diagnosis were 5 years or older. In contrast, genetic diagnoses were achieved in only 4% (7/179) of patients under 5 years old. They attribute this low diagnostic yield to the multifactorial nature of ASD, where both common and rare variants contribute to the risk of autism [14,28,29,30,31,32].

Following this line, our study showed a diagnostic yield of 66.7% in patients older than 5 years compared to 33.3% in younger children. However, these differences did not reach statistical significance (66.7% vs. 33.3%, *p =* 0.060), possibly due to the small sample size.

Thus, our results highlight the limited usefulness of genetic studies in the pediatric population under 5 years of age due to the difficulty in diagnosis. In our study, 19% of patients were younger than 5 years, which may have influenced our overall diagnostic yield. If more studies corroborate this finding, it could lead to a change in clinical management by establishing an age limit for requesting genetic studies in patients with ASD.

A recent study showed that the diagnostic yield of proband-only exome sequencing was slightly lower than that of proband–parents trio studies, reaching about 25% [33]. In our study, the overall diagnostic rate for proband ES was 12.5% (22 of 176). After the reclassification of genetic variants through parental analysis (where possible), the diagnostic yield increased by 4.6%, resulting in a final diagnostic rate of 17.1%.

In our study, the diagnostic yield in the isolated intellectual disability group was 26.6% (16/60). The causal diagnostic yield was significantly higher in the group of syndromic patients (70.6% vs. 29.4%; *p =* 0.036), consistent with findings in the literature [9,33,34]. Therefore, this underscores the importance of a comprehensive medical history and physical examination.

With the affordability and low incidence of incidental findings, disease gene panels are widely utilized in clinical practice for diagnosing heterogeneous disorders. We identified incidental findings in 11.9% of cases, unrelated to the initial reason for the study. Among these, 10.7% were carrier states of diseases with autosomal recessive inheritance, and actionable genes related to cancer were detected in two cases: *BRCA2* gene (associated with increased susceptibility to breast and ovarian cancer AD) and *BRIP1* gene (moderate risk of ovarian cancer with incomplete penetrance) [35,36]. In both cases, these findings were reported, and a multidisciplinary action protocol was established. The parents signed an informed consent where they authorized knowing incidental findings according to the recommendations of the American College of Medical Genetics and Genomics (ACMG) [37] in both cases. Both patients were referred to a family cancer department, where they received genetic counseling.

### Limitations

Some limitations of our study include the small sample size and the lack of segregation analysis in cases where parental analysis was not possible, which could influence the profitability analysis. A prospective study with a larger sample size will be necessary to confirm these findings. On the other hand, we must consider that segregation analysis does not always allow for the reclassification of variants, as its result is a moderate classification criterion without confirmed paternity.

Although a sequential approach using exome sequencing (ES) through targeted gene panels of only probands is 2–3 times cheaper than trio analysis, it presents significant drawbacks. Firstly, it requires more time to obtain results, which may delay clinical diagnosis and, consequently, the initiation of appropriate therapeutic interventions. Furthermore, this approach places an additional burden on clinicians, who must explain non-diagnostic or inconclusive results to families, request further testing from the parents, and reinterpret results as new information becomes available. This not only increases administrative workload but also adds uncertainty for patients and families, prolonging the diagnostic process and complicating clinical decision making [9].

However, the identification of disease-associated genes can significantly improve the understanding of disease pathogenesis and trajectories. There are already some studies that are beginning to talk about precision medicine in NDDs, which is a great hope in the near future [38]. Moreover, genetic counseling plays a crucial role in the decision making regarding reproductive planning [12].

## 5. Conclusions

The diagnostic yield from exome sequencing (ES) using targeted gene panels was lower than that reported in other studies. In our cohort, we identified disease-causing or likely pathogenic variants in 12.5% of the tested individuals, increasing to 17.1% after incorporating parental studies in selected cases. This sequential approach, adding parental testing, improved the diagnostic yield by 4.6%. Notably, ES demonstrated a lower diagnostic yield in patients under five years of age, while the yield was higher in syndromic patients within the ID/GDD group.

Although trio-based whole exome sequencing offers the highest diagnostic yield, its high cost limits its widespread use in clinical settings. In contrast, when this is not possible, ES using targeted panels in the proband offers a practical and cost-effective solution for diagnosing neurodevelopmental disorders (NDDs), while still allowing for the identification of pathogenic variants. Furthermore, parental testing improves diagnostic accuracy, particularly in cases involving variants of uncertain significance (VUS), thereby contributing to a deeper understanding of NDDs and enhancing the clinical utility of genetic testing.

## Figures and Tables

**Table 1:** Clinical characteristics of patients.

Age (Mean; Range)	6 Years (9 m–16 y)
Male sex, n (%)	127 (72.1%)
Neurodevelopmental disorder, n (%): ID/GDD ASD ID/GDD + ASD	67 (38.1%) 62 (35.2%) 47 (26.7%)
Ethnicity, n (%) Spanish Arab South American Other	139 (79%) 16 (9%) 14 (8%) 7 (4%)
Mother’s age (mean)	32 years
Father’s age (mean)	35 years
Consanguinity, n (%)	6 (3.4%)
Family history of ID and/or ASD, n (%)	33 (18.8%)
Pregnancy, n (%) Spontaneous Assisted reproductive therapy	164 (93.2%) 12 (6.8%)
Gestational age, n (%) Term deliveries Premature deliveries >32 weeks <32 weeks	155 (88%) 21 (12%) 4 (18.7%) 17 (81.3%)
Delivery Eutocic/scheduled cesarean section Instrumental delivery/urgent cesarean section	136 (77.2%) 40 (22.8%)

**Table 2:** Diagnostic yield by groups before and after studying parental segregation.

Diagnostic Yield by WES in Proband (n = 176)			
ID/GDD group (n = 67)	ASD group (n = 62)	ID/GDD +ASD (n = 47)	Global
20.8%	3.2%	12.7%	12.5%
Diagnostic Yield after Studying Parental Segregation (n = 152)			
ID/GDD group (n = 60)	ASD group (n = 53)	ID/GDD +ASD (n = 39)	Global
26.6%	1.8%	23%	17.1%

ID: intellectual disability; ASD: autism spectrum disorder; and GDD: global developmental delay.

**Table 3:** Pathogenic and likely pathogenic variants after parental confirmation via Sanger.

ID	Gene	Location	DNA Variants	Protein Variants	Zygosity	Inheritance	OMIM#	De Novo
P1	*HIVEP2*	6q24.2	c.737_740dup	p.(Val248IlefsTer11)	Het	AD	#616977	de novo
P10	*CHAMP1*	13q34	c.1036G>T		Het	AD	#616579	NA
P28	*HUWE1*	Xp11.22	c.12374A>G	p.(Glu4125Gly)	Hem	*XL*	#30959	de novo
P40	*GATA3*	10p14	c.924+1G>A		Het	AD	#131320	de novo
P50	*KDM6B*	17p13.1	c.1180C>G	p.(Pro394Ala)	Het	*AD*	#618505	de novo
P51	*TLK2*	17q23.2	c.195_199del,	p.(Arg65SerfsTer2)	Het	AD	#618050	NA
P60	*CNKSR2*	Xp22.12	c.352C>T	p.(Arg118Ter)	Het	XL	#301008	NA
P68	*MED13L*	12q24.21	c.1280+1G>T		Het	AD	#616789	de novo
P69	*SHANK3*	22q13.33	c.3525delG	p.(Asp1176ThrfsTer4	Het	AD	#606230	de novo
P71	*CACNA1C*	12p13.33	c.1113+5G>A		Het	AD	#601005 #618447	de novo
P80	*POGZ*	1q21.3	c.2171dup	p.(Leu725SerfsTer19)	Het	AD	#614787	NA
P89	*SOX5*	12p12.1	c.931+5G>		Het	AD	#616803	de novo
P107	*CUL3*	2q36.2	c.802delC	p.(Leu268SerfsTer5)	Het	AD	#619239	de novo
P111	*NFIX*	19p13.13	c.583+1G>A		Het	AD	#614753	de novo
P123	*CDKL5*	Xp22.13	c.1675C>T	p.(Arg559Ter)	Het	XL	#300672	NA
P128	*SPAST*	2p22.3	c.1321+1G>A		Het	AD	#604277	NA
P135	*CIC*	19.13.2	c.1331G>T	p.(Cys444Phe)	Het	AD	#617600	de novo
P137	*DDX3X*	Xp11.4	c.1770-3_1770-2dup		Het	XL	#300958	NA
P144	*ASH1L*	1q22	c.4025G>A	p.(Arg1342Gln)	Het	AD	#617796	de novo
P169	*CDH23*	10q22.1	c.4021G>A c.5735G>A	p.(Asp1341Asn) p.(Arg1912Gln)	Het	AR	#601067	Maternal Paternal

Het = heterozygous; Hem = hemizygous; and NA = segregation information not available.

**Table 4:** Chromosomal CNVs detected by exome sequencing.

ID	Deletion/Duplication	Location	Estimated Size	Reported Critical Genes	CNV
P6	Deletion	1q44	1.5 Kb	*ZBTB18*	#612337
P22	Duplication	9p24	192 Kb	*DOCK8*	#614113
P27	Deletion	7q21.3	8.7 Mb	*COL1A2*	
P48	Duplication	2p16.1p11.2	32.6 MB	*BCL11A*	
P148	Deletion	22q11.2	1.9 Mb	-	#611867
P176	Duplication	16p11.2	529 kb	-	#614671

## Data Availability

The original contributions presented in the study are included in the article, further inquiries can be directed to the corresponding author.
